# Ecliptasaponin A alleviates inflammation and fibrosis in experimental MASH mice via targeting the NLRP3 inflammasome and YAP signaling pathway

**DOI:** 10.1186/s13020-025-01321-9

**Published:** 2026-01-13

**Authors:** Kai Gao, Wei Zhang, Meina Zhao, Dong Xu, Xingru Tao, Chao Guo, Yang Du, Fuxing Jin, Wangting Li, Meiyou Liu, Yunyang Lu, Jingwen Wang

**Affiliations:** 1 Department of Pharmacy, Xijing Hospital, Fourth Military Medical University, Xi’an, 710032 China; 2 Department of Chinese Materia Medica and Natural Medicines, School of Pharmacy, Fourth Military Medical University, Xi’an, 710032 China; 3 Research Institution, Xijing Hospital, Fourth Military Medical University, Xi’an, China

**Keywords:** MASH, Ecliptasaponin A, NLRP3 inflammasome, YAP

## Abstract

**Background:**

Metabolic dysfunction-associated steatohepatitis (MASH) has emerged as the primary contributor to the increasing incidence and mortality rates linked to cirrhosis and hepatocellular carcinoma globally, while the availability of clinical treatment drugs remains severely limited. Ecliptasaponin A (EA), naturally isolated from Ecliptae Herba, possesses multiple biological activities. However, the effects of EA on MASH remain unclear.

**Purpose:**

This study aimed to explore the roles of EA in MASH and its engaged mechanisms.

**Methods:**

Two established NASH animal models, non-obese MASH induced by methionine-choline-deficient (MCD) dietary administration and obese MASH developed through high-fat/high-cholesterol (HFHC) feeding were employed to assess EA's therapeutic effects in vivo. RNA-seq analysis was conducted to uncover EA's molecular mechanisms. Complementary in vitro investigations utilized LPS-treated BMDMs and THP1 cells, and TGF-β1-activated LX-2 hepatic stellate cells to systematically examine EA's cellular-level impacts and regulatory pathways.

**Results:**

Oral administration of EA demonstrated dose-responsive therapeutic effects against MCD/HFHC-induced MASH. The compound effectively attenuated hepatic steatosis, inflammatory responses, and fibrotic progression in experimental models through dual modulation of NLRP3 and YAP signaling pathways. Mechanistic studies revealed EA specifically suppressed NLRP3 inflammasome activation in BMDMs without affecting AIM2 or NLRC4 inflammasomes, effectively blocking cytokine secretion, pyroptotic cell death, caspase-1 activation, and inflammasome complex formation. Molecular interactions analysis confirmed EA directly binds to NLRP3, disrupting inflammasome assembly. In LX-2 cells, EA suppressed TGF-β1-induced COL1A1 and α-SMA expression while reducing YAP protein levels. Genetic silencing or pharmacological inhibition of YAP failed to potentiate EA's anti-fibrotic effects on α-SMA suppression, Collagen I expression, or YAP-regulated gene transcription. Molecular docking and SPR showed that EZ could directly bind to NLRP3 and YAP.

**Conclusion:**

These findings reveal novel perspectives on the natural compound Ecliptasaponin A, demonstrating its dual-targeting capability against both NLRP3 inflammasome activation and YAP signaling cascades. This discovery highlights its potential as a promising therapeutic agent for mitigating MASH.

**Supplementary Information:**

The online version contains supplementary material available at 10.1186/s13020-025-01321-9.

## Introduction

Metabolic dysfunction-associated steatotic liver disease (MASLD; previously termed non-alcoholic fatty liver disease, NAFLD) has emerged as the predominant chronic hepatic condition globally, affecting approximately one-quarter of adults worldwide, while demonstrating strong bidirectional correlations with metabolic syndrome components [6, 7, 28]. Metabolic dysfunction-associated steatohepatitis (MASH), formerly known as NASH, originates from MASLD progression, with approximately one-fifth of MASLD patients advancing to MASH characterized by hepatic inflammation, cellular damage, and most critically, fibrotic progression [23]. This condition has surpassed viral hepatitis as the primary contributor to escalating global health burdens from cirrhosis and hepatocellular carcinoma [27, 31]. Currently, Resmetirom stands as the only FDA-approved pharmacological intervention for MASH [18]. However, its prohibitive cost and limited accessibility in the United States restrict therapeutic alternatives.

Hepatic inflammation and fibrosis represent pivotal processes driving the progression of MASH. Recent studies highlight the NLRP3 inflammasome's critical involvement in MASH pathogenesis, particularly through its role in metabolic inflammation regulation [37]. This intracellular protein complex facilitates IL-1β maturation and activation, with elevated caspase-1-dependent IL-1β release observed in MASH patients. Experimental models demonstrate that NLRP3 deficiency confers protection against diet-induced hepatic steatosis in MASLD mice, reinforcing its pathogenic significance [29, 30, 36]. While predominantly expressed in hepatic immune cells such as Kupffer cells and macrophages, NLRP3 shows minimal activity in parenchymal cells like hepatocytes and HSCs [25, 34]. Clinical evidence reveals substantial increases in caspase-1 and IL-1β activation correlating with NASH progression, where inflammasome triggering exacerbates lipid accumulation, inflammatory responses, and fibrotic transformation [10, 25, 36]. The Hippo pathway component YAP interacts with TAZ to regulate HSC-derived myofibroblast activity, with emerging data showing its role in glutamine metabolism modulation and HSC activation during MASLD development [3–5, 24, 38]. Pharmacological YAP inhibition demonstrates therapeutic potential through HSC deactivation and fibrosis regression [12]. Thus, targeting NLRP3 inflammasome and YAP signaling pathway are an effective strategy in pharmacotherapy of MASH.

Ecliptasaponin A (EA), a principal bioactive component of pentacyclic triterpenoid saponins derived from *Eclipta prostrata* (Linn.), has recently shown remarkable therapeutic potential in inflammation-associated pathologies. Emerging evidence from murine models indicates EA's efficacy in mitigating pulmonary fibrosis, renal fibrosis, and osteoarthritis through its anti-inflammatory mechanisms [14, 22, 40]. Given the pathophysiological triad of hepatic steatosis, inflammatory response, and fibrotic progression characterizing metabolic dysfunction-associated steatohepatitis (MASH), we hypothesized EA's therapeutic applicability in this condition. To our knowledge, no prior studies have explored EA's impact on MASH pathogenesis. Our investigation employed two distinct dietary models—methionine-choline deficient (MCD) diet-induced non-obese MASH and high-fat high-cholesterol (HFHC) diet-induced obese MASH—to comprehensively evaluate EA's pharmacological effects. Through RNA sequencing (RNA-seq) analysis of hepatic tissues combined with mechanistic validation in both in vivo and in vitro systems, we identified novel therapeutic targets. Our results provided the first confirmation that EA targeted the NLRP3 inflammasome and YAP signaling pathway, thereby exerting anti-inflammatory and anti-fibrotic effects. This study not only proposes a novel therapeutic strategy for MASH intervention but also establishes a mechanistic foundation for understanding EA's multi-target pharmacological effects in hepatic pathophysiology.

## Materials and methods

### Mice models

Male C57BL/6 mice (18–20 g body weight) were obtained from the Animal Research Center at Fourth Military Medical University. These animals were maintained in pathogen-free conditions with controlled environmental parameters (22 ± 2 °C ambient temperature, 12-h photoperiod cycle). Following the initial week of adjustment, mice were subjected to a range of therapies. All procedures received ethical approval from the Institutional Animal Ethics Committee of Fourth Military Medical University (Approval ID: KY20231295), complying with national guidelines for laboratory animal welfare.

To establish metabolic dysfunction-associated steatohepatitis (MASH) models, the mice were fed the MCD diet for 8 weeks or HFHC (40 kcal% fat, 20 kcal% fructose and 2% cholesterol; Synergy Biology, Nanjing, China) for 24 weeks. Ecliptasaponin A (EA, CAS: 78285-90-2, Purity: 98%) was purchased from Baoji Chengguang Biotechnology Co., Ltd. (Shaanxi, China). The animals were divided into six experimental cohorts of 6 animals through randomization: control, model, EA-L (10 mg/kg/d), EA-M (20 mg/kg/d), EA-H (40 mg/kg/d), and Resmetirom (15 mg/kg/d). From the fifth or thirteenth week until the conclusion of the experiment, the subjects received various doses of EA, positive controls, and normal saline via daily gavage in the MCD or HFHC diet. Plasma and liver tissues were collected from the mice that were euthanized at the conclusion of the experiment.

### Cell culture and treatments

Primary bone marrow-derived macrophages (BMDMs) and primary mouse Kupffer cells (KCs) were isolated from 8 to 10 weeks age of male wild-type C57BL/6 mice. BMDMs were maintained in DMEM supplemented with 10% fetal bovine serum, 50 ng/ml murine macrophage colony-stimulating factor (M-CSF), and 1% penicillin/streptomycin (P/S). THP1 cells were cultured in RPMI- 1640 medium supplemented with 10% FBS and 1% P/S. LX-2 cells were cultured in DMEM containing 10% FBS and 100 U/ml penicillin–streptomycin solution. After growing to 70–80% confluence, LX-2 cells underwent 6 h of incubation in serum-free medium, followed by 24 h of treatment using 10 ng/ml TGF-β1 and EA (5, 10, and 20 μM). All cell lines were grown in a 5% CO_2_ incubator at 37 °C. EA was dissolved in the corresponding cell culture medium with DMSO as a solvent, resulting in a final DMSO concentration of 0.1%.

### Cell viability assay

Cell viability was assessed using CCK-8 assay. Cells were seeded at a density of 1 × 10^4^ cells per well in a 96-well plate and cultured overnight. Subsequently, the culture medium was replaced with fresh medium supplemented with varying concentrations of EA (0, 5, 10, 20, 40 μM), and the cells were further incubated for 24 h. Following treatment, 10 μL of CCK-8 solution was added to each well, and the plates were incubated in the dark at 37 °C for 2 h. Absorbance was measured at 450 nm using a microplate reader.

### Inflammasome activation

Macrophages were primed by treatment with 50 ng/ml (BMDM) or 20 ng/ml (KC) of LPS for 4 h. Following this, the culture medium was changed to Opti-MEM containing bioactive compounds. After 1 h of incubation, NLRP3 activation was typically induced by one of the following treatments: 5 mM ATP for 45 min, 7.5 μM nigericin for 30 min, 200 μg/mL SiO_2_ for 6 h, or transfection with 1 μg/mL poly (I: C) using Lipofectamine 2000 for 6 h. To activate the NLRC4 or AIM2 inflammasomes, Salmonella infection or transfection with 1 μg/mL poly (dA: dT) using Lipofectamine 2000 was conducted for 6 h.

### Nuclear protein and cytoplasmic protein extraction

Nuclear and cytoplasmic proteins were isolated using the Nuclear and Cytoplasmic Protein Extraction Kit (KGP150/KGP1100) provided by KeyGEN BioTECH. LX-2 cells were grown and maintained in 10 cm culture dishes under standard conditions. After reaching the desired confluence, cells were harvested by centrifugation at 800 × g for 3 min and washed twice with ice-cold phosphate-buffered saline (PBS). The cell pellet was resuspended in a mixture of 450 µL cold Buffer A and 50 µL Buffer B, followed by incubation on ice for 30 min. Buffer A was freshly prepared by adding 17 µL of PMSF (100 mM) and 1 µL of protease inhibitor to 1 mL of the base buffer. Following centrifugation at 3,000 rpm for 10 min at 4 °C, the supernatant containing cytoplasmic proteins was collected. The remaining pellet was then suspended in 100 µL of ice-cold Buffer C, which was similarly supplemented with 17 µL PMSF (100 mM) and 1 µL protease inhibitor per mL. The suspension was vortexed vigorously for 15 s and incubated on ice for 45 min, with additional 15-s vortexing steps every 10 min. After a final centrifugation at 14,000 × g for 30 min at 4 °C, the supernatant was collected as the nuclear protein fraction. Both nuclear and cytoplasmic extracts were processed further according to subsequent experimental requirements.

### Cell migration assays

Transwell assays were performed to assess cell migration by seeding LX2 cells (2 × 10^5^ cells in 200 µL) into the upper chamber of a 24-well polycarbonate transwell insert with an 8 µm pore size (Corning Incorporated), using medium containing 5% serum. The lower chamber was filled with 700 µL of medium supplemented with 10% FBS to serve as a chemoattractant. Following 24 h of incubation, the migrated cells were fixed with 4% paraformaldehyde and stained with 0.1% crystal violet solution. Non-migrated cells on the upper surface of the membrane were carefully removed with a cotton swab. Migrated cells on the lower surface were visualized and imaged using an inverted microscope. Through parallel experiments conducted under identical cell density, treatment factors, and culture conditions, the cell viability of each experimental group was assessed in 6-well plates to verify consistency in cell viability across all conditions used in the migration assay.

### Cell transfection and inhibitors

siRNA of YAP was transfected to LX-2 cells using Lipofectamine^™^ 3000 Transfection Reagent (Thermo Fisher). The YAP inhibitor Verteporfin was purchased from Topscience Biotechnology (Shanghai, China).

### Liver histological observation

Processed liver tissues were dried, clarified, and embedded in paraffin. For light microscopy, hematoxylin and eosin (H&E), Oil Red O, and Masson's trichrome staining were applied to the tissue sections of these organs.

### qRT-PCR analysis

After TRIzol^®^ reagent (Life Technology, NY, USA) was used to isolate total RNA from frozen liver samples of mice, the resulting RNA was purified and reverse-transcribed using a PrimeScript^™^ RT reagent Kit with gDNA Eraser (Takara Bio, Shiga, Japan). A ViiA 7 Real-Time (RT) PCR System (Applied Biosystems, CA, USA) was used to perform RT-PCR using TB Green Premix Ex Taq II (Takara Bio, Shiga, Japan). The quantity of messenger RNA (mRNA) was measured using the comparative CT technique and normalized to that of GAPDH.

### Western-blot analysis

Total, cytoplasmic, and nuclear protein levels in liver tissues were quantified after extraction. Proteins were subsequently extracted following the manufacturer's instructions. Protein samples were then prepared by heat denaturation at 100 °C for 10 min. Separation was performed using 10% SDS-PAGE, followed by transfer of proteins onto PVDF membranes. The membranes were blocked with blocking solution at room temperature for 2 h and washed three times with PBS. Primary antibodies against YAP (Abcam, ab205270, 1:1000), COL1A1 (Abcam, ab21286, 1:1000), α-SMA (Proteintech, 14395-1-AP, 1:2000), TAZ (Abcam, ab224239, 1:1000), NLRP3 (Proteintech, 30109-1-AP, 1:2000), Caspase 1 p20 (Proteintech, 22915-1-AP, 1:2000), Caspase 1 p45 (Proteintech, 84735-1-RR, 1:5000), pro-IL-1β (R&D, AF-401-NA, 1:1000), cleaved IL-1β (Cell Signaling Technology, 12242, 1:2000), ASC (Abcam, ab309497, 1:1000) and GAPDH (Proteintech, 60004-1-Ig, 1:5000) were applied, and membranes were incubated overnight at 4 °C. After washing for 30 min, the membranes were incubated with horseradish peroxidase-conjugated anti-rabbit IgG secondary antibody for 2 h. Finally, protein bands were visualized using ECL chemiluminescent substrate, and images were captured and analyzed quantitatively using imaging software.

### Molecular docking

Proteins in the Omic analysis and experimental verification were chosen as potential targets for molecular docking investigations. Protein Data Bank (PDB) files for primary targets were sourced from the RCSB database and the 3D structures of the active compounds in Panax ginseng were obtained from the PubChem database. Core target proteins were prepared by removing the solvent molecules and performing hydrogenation and charging using AutoDock Tools 1.5.6. Both proteins and active compounds were saved in "pdbqt" format for docking, using AutoDock Vina to establish the docking box’s dimensions and position.

### Target validation by SPR analysis

Surface plasmon resonance (SPR) experiments were performed to assess bioactive compound-protein interactions. Test substances were prepared in a concentration gradient ranging from 3.125 to 200 μM (3.125, 6.25, 12.5, 25, 50, 100, 200 μM) utilizing a PBS-P buffer containing 5% DMSO. The system maintained a constant flow rate of 30 μl/min during operation, with binding phase duration of 60 s followed by a 120-s dissociation period. Subsequent data processing and affinity curve generation were conducted through the Biacore T200 evaluation suite.

### Statistical analysis

Quantitative findings are displayed as average values ± standard deviations. Analytical procedures were executed through GraphPad Prism 9.4 software, implementing one-way ANOVA followed by Bonferroni's post hoc test for multiple group analyses. A significance threshold of p < 0.05 was established for determining statistical differences.

## Results

### EA mitigated hepatic steatosis, inflammation, and fibrosis induced by the MCD diet

**Fig. 1:**
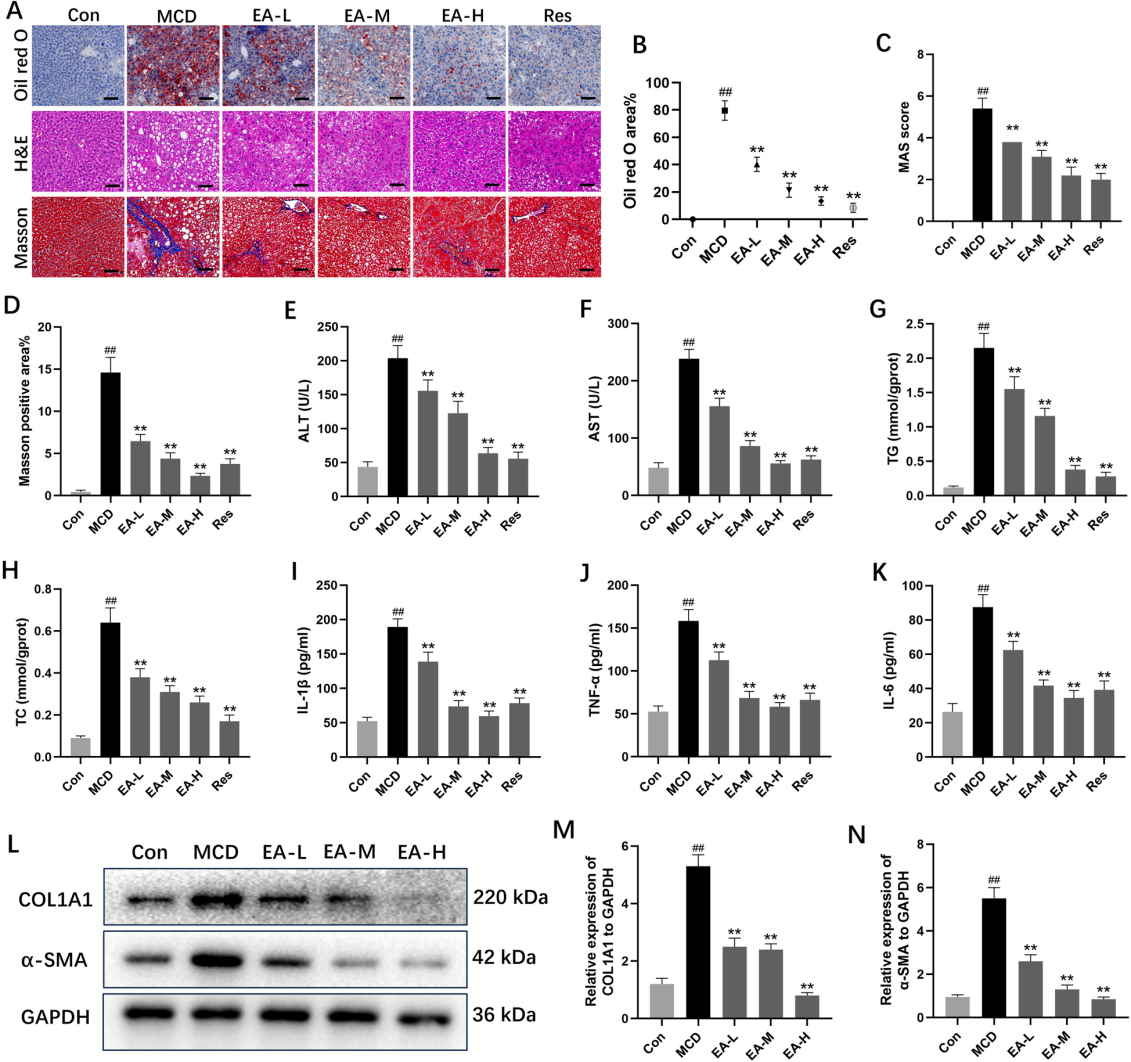
EA suppressed hepatic steatosis, inflammation and fibrosis in MCD-induced MASH. **A** Representative oil red O staining, H&E staining and Masson staining pictures (the scale bars represent for 50 μm) of the livers of mice from different groups, n = 6. **B** Oil red O per area, n = 6. **C** The double-blind assessment of hepatic pathological injury utilizing the MAS system, n = 6. **D** Quantification of the Masson positive area, n = 6. **E** and **F** Serum AST and ALT, n = 6. **G**, **H** The contents of hepatic TG and TC, n = 6. **I**–**K** The content of plasma inflammatory cytokines after feeding with MCD diets, n = 6. **L**–**N** Representative western blot analysis of fibrosis-associated factors in liver tissues, n = 3. Data were presented as the mean ± SD. ^##^*P* < 0.01 vs. control; ^**^P < 0.01 vs. MCD

Mice were subjected to an MCD diet for 4 weeks, followed by administration of varying doses of EA (10, 20, and 40 mg/kg/d) for an additional 4 weeks. As illustrated in Fig. 1E–H, levels of serum AST, ALT, and intrahepatic TG, TC were significantly elevated in the MCD-induced model group compared to the control group, while EA effectively reduced these levels in a dose-dependent manner. Furthermore, histological assessments using oil red O, H&E, and Masson staining revealed that EA-treated mice exhibited less severe steatosis, reduced inflammatory cell infiltration, and diminished collagen deposition compared to MCD mice (Fig. 1A–D). A double-blind evaluation utilizing the MAFLD activity score system (MAS) indicated the presence of hepatic steatosis, lobular inflammation, and hepatocellular ballooning in mice administered MCD (Fig. 1C). The MAS score and levels of pro-inflammatory markers, including IL-6, IL-1β, and TNF-α, were significantly decreased following EA administration, demonstrating the compound's efficacy in attenuating hepatic steatosis and inflammation in a dose-responsive manner (Fig. 1I–K). Additionally, EA reduced the protein expression of fibrosis markers, α-SMA and COL1A1, which had been significantly elevated in the model group (Fig. 1L–N). Collectively, these findings suggest that EA treatment confers protection against hepatic lipid dysfunction, inflammation, and fibrosis induced by the MCD diet.

### EA alleviated hepatic steatosis, inflammation, and fibrosis induced by the HFHC diet

**Fig. 2:**
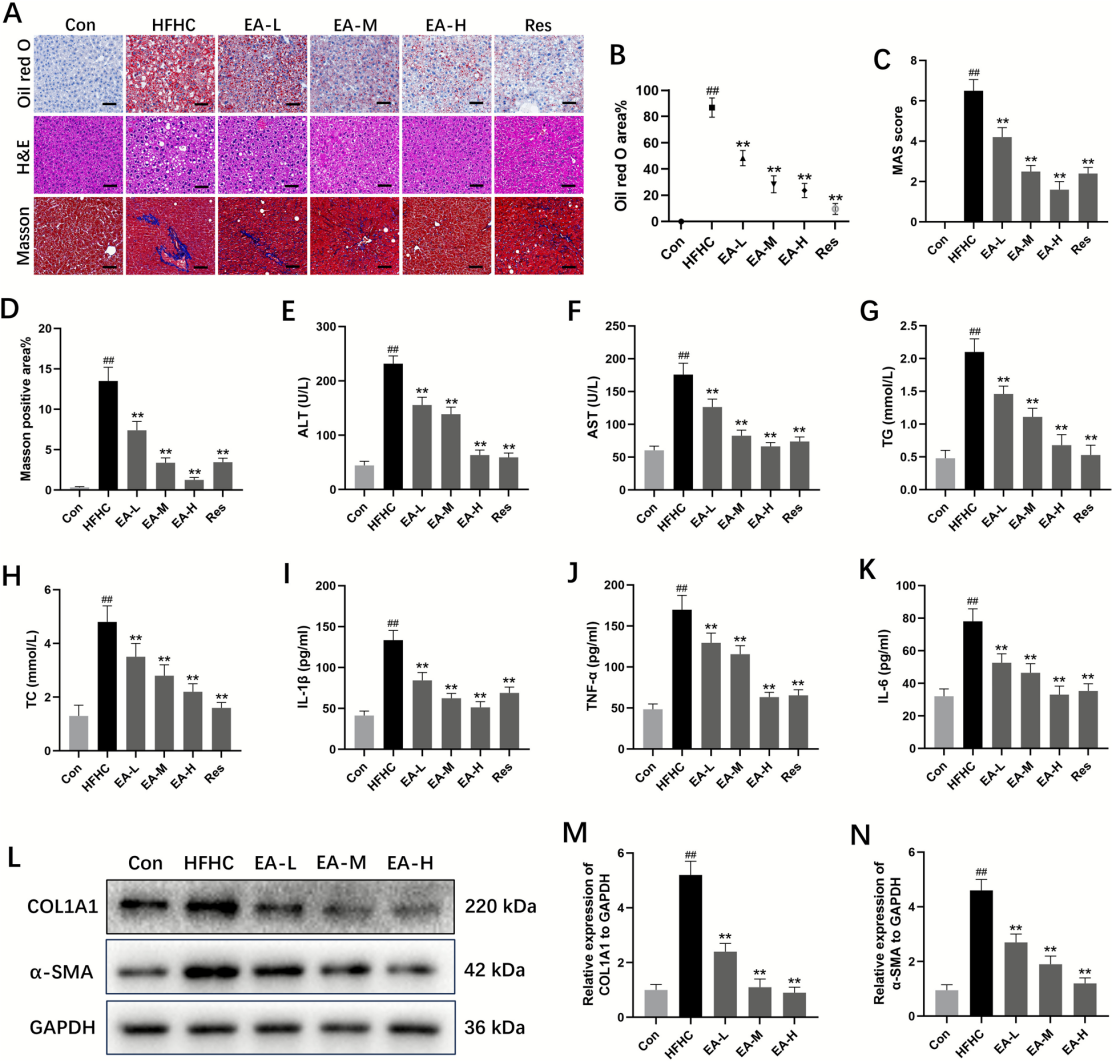
EA alleviated hepatic steatosis, inflammation and fibrosis in HFHC-induced MASH. **A** Representative oil red O staining, H&E staining and Masson staining pictures (the scale bars represent for 50 μm) of the livers of mice from different groups, n = 6. **B** Oil red O per area, n = 6. **C** The double-blind assessment of hepatic pathological injury utilizing the MAS system, n = 6. **D** Quantification of the Masson positive area, n = 6. **E**–**H** Serum TG, TC, AST and ALT, n = 6. **I**–**K** The content of plasma inflammatory cytokines after feeding with HFHC diets, n = 6. **L**–**N** Representative western blot analysis of fibrosis-associated factors in liver tissues, n = 3. Data were presented as the mean ± SD. ^##^*P* < 0.01 vs. control; ^**^P < 0.01 vs. HFHC

To further elucidate the protective effects of EA against MASH in vivo, we established an HFHC diet-induced MASH model characterized by severe hepatic steatosis, inflammation, and fibrosis, ultimately leading to steatohepatitis with pathological features akin to human MASH. Mice were fed an HFHC diet for 12 weeks and subsequently administered varying doses of EA (10, 20, and 40 mg/kg/d) for an additional 12 weeks. The results were consistent with those of the MCD model experiment. Serum levels of AST, ALT, TG, and TC were significantly higher in the HFHC-induced model group compared to the control group, and EA effectively reduced these levels in a dose-dependent manner (Fig. 2E–H). Furthermore, histological assessments using oil red O, H&E, and Masson staining revealed that EA-treated mice exhibited less severe steatosis, reduced inflammatory cell infiltration, and diminished collagen deposition compared to HFHC mice (Fig. 2A–D). The levels of pro-inflammatory markers, such as IL-6, IL-1β, and TNF-α, were significantly reduced after EA administration, confirming the compound's ability to mitigate hepatic steatosis and inflammation in a dose-responsive manner (Fig. 2I–K). Moreover, EA similarly reduced the protein expression of early pro-fibrogenic markers α-SMA and COL1A1, which had been markedly elevated in the model group (Fig. 2L–N). These findings confirm the role of EA in attenuating HFHC diet-induced hepatic steatosis, inflammation, and fibrosis progression.

### The effect of EA on MASH was related to NLRP3 and YAP signaling pathways

**Fig. 3:**
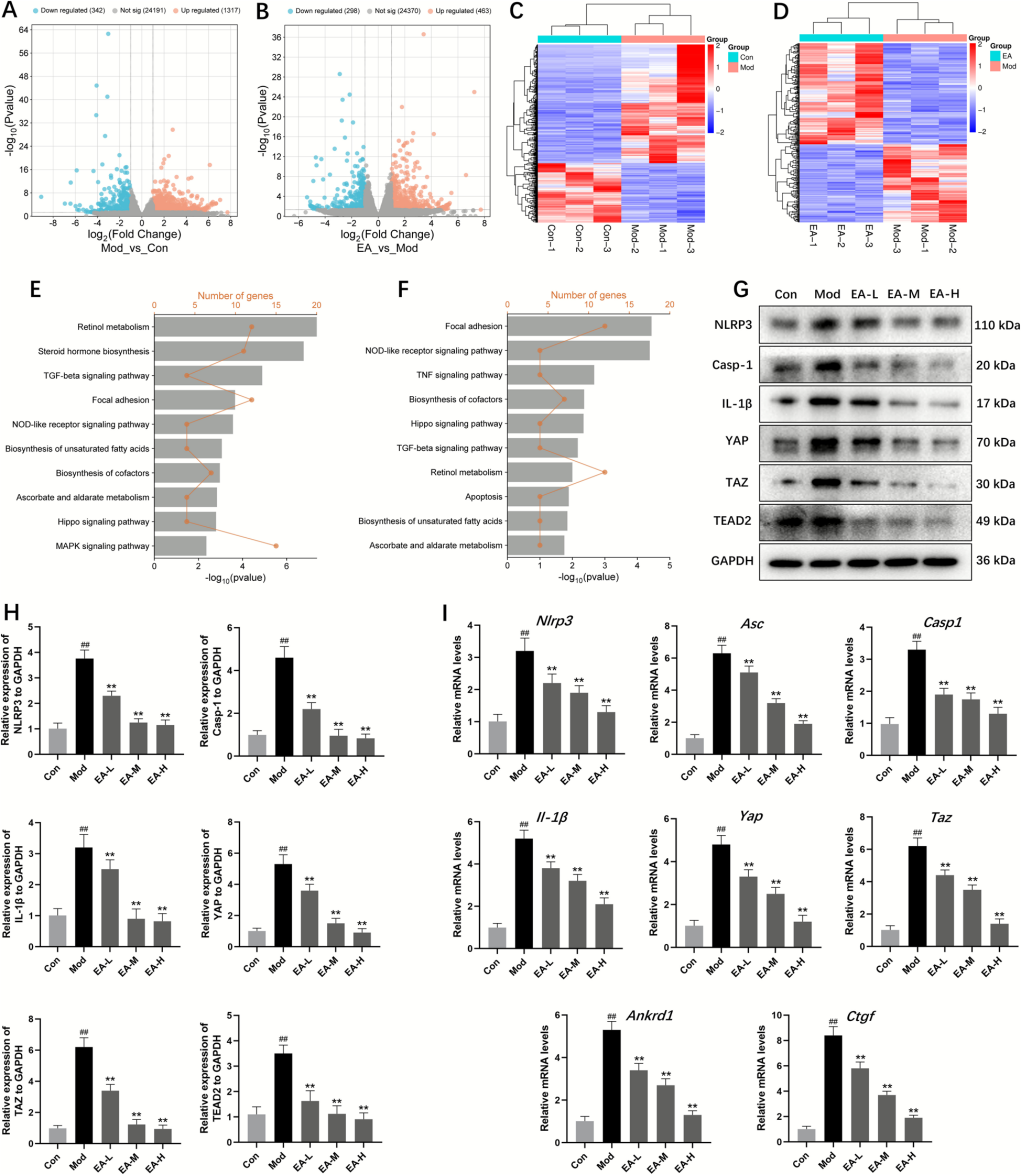
EA mitigated HFHC diet-induced MASH through the modulation of NLRP3 inflammasome and YAP signaling pathway. **A** and **C** Volcano plot and heatmap of DEGs between control group and HFHC group in RNA-seq. **B** and **D** Volcano plot and heatmap of DEGs between EA group and HFHC group in RNA-seq. **E** KEGG enrichment of DEGs in the HFHC group in comparison with the control group. **F** KEGG enrichment of DEGs in the EA group in comparison with the HFHC group. **G** and **H** Western blotting of proteins involved in NLRP3 inflammasome and YAP signaling pathway in HFHC-fed mice. **I** Quantitative real-time PCR analysis of the transcript levels of genes related to NLRP3 inflammasome and YAP signaling pathway. Data were presented as the mean ± SD (n = 3). ^##^*P* < 0.01 vs. control; ^**^P < 0.01 vs. HFHC

To investigate the molecular mechanisms of EA in MASH progression, hepatic RNA sequencing was conducted using mice maintained on an HFHC dietary regimen. Comparative analysis revealed 1659 differentially expressed genes (DEGs) between model and control groups, with 1317 demonstrating elevated expression and 342 showing reduced expression. Subsequent evaluation of EA-H treatment (40 mg/kg) versus the model group identified 761 DEGs, including 463 upregulated and 298 downregulated transcripts, as visually represented through volcano plots and hierarchical clustering diagrams (Fig. 3A–D). Functional annotation through KEGG pathway analysis demonstrated significant enrichment patterns: model group upregulations and EA-H group downregulations were predominantly associated with NOD-like receptor signaling and Hippo-mediated transduction cascades (Fig. 3E, F). These findings strongly implicate NLR and Hippo pathway modulation as critical mechanisms underlying EA's therapeutic efficacy against HFHC-induced MASH. The NLRP3 inflammasome-mediated inflammation and YAP-driven activation of hepatic stellate cells are key pathological mechanisms closely associated with MASH, which this study specifically targets. Therefore, we prioritize in-depth investigation of NLRP3 and YAP.

To thoroughly investigate the signaling pathways regulated by EA, we examined the expression of genes and proteins linked to NOD-like receptor (NLR) and Hippo signaling pathways in liver tissues using western blotting and qRT-PCR. Compared to the control group, the model group exhibited a significant increase in the protein levels of NLRP3, Casp1, IL-1β, YAP, TAZ, TEAD2. Conversely, EA therapy reduced NLRP3, Casp1, IL-1β, YAP, TAZ, and TEAD2 expression (Fig. 3G and H). Additionally, gene expressions with inflammation (*Nlrp3*, *Asc*, *Casp1* and* Il-1β*), and fibrosis (*Yap*, *Taz*, *Ankrd1* and *Ctgf*) were elevated. However, EA administration resulted in the suppression of gene expressions (Fig. 3I). These findings suggest that EA effectively ameliorates inflammation and fibrosis in MASH mice by NLRP3 inflammasome and YAP/TAZ signaling pathway.

### EA inhibited NLRP3 inflammasome activation in BMDMs and THP1 cells

**Fig. 4:**
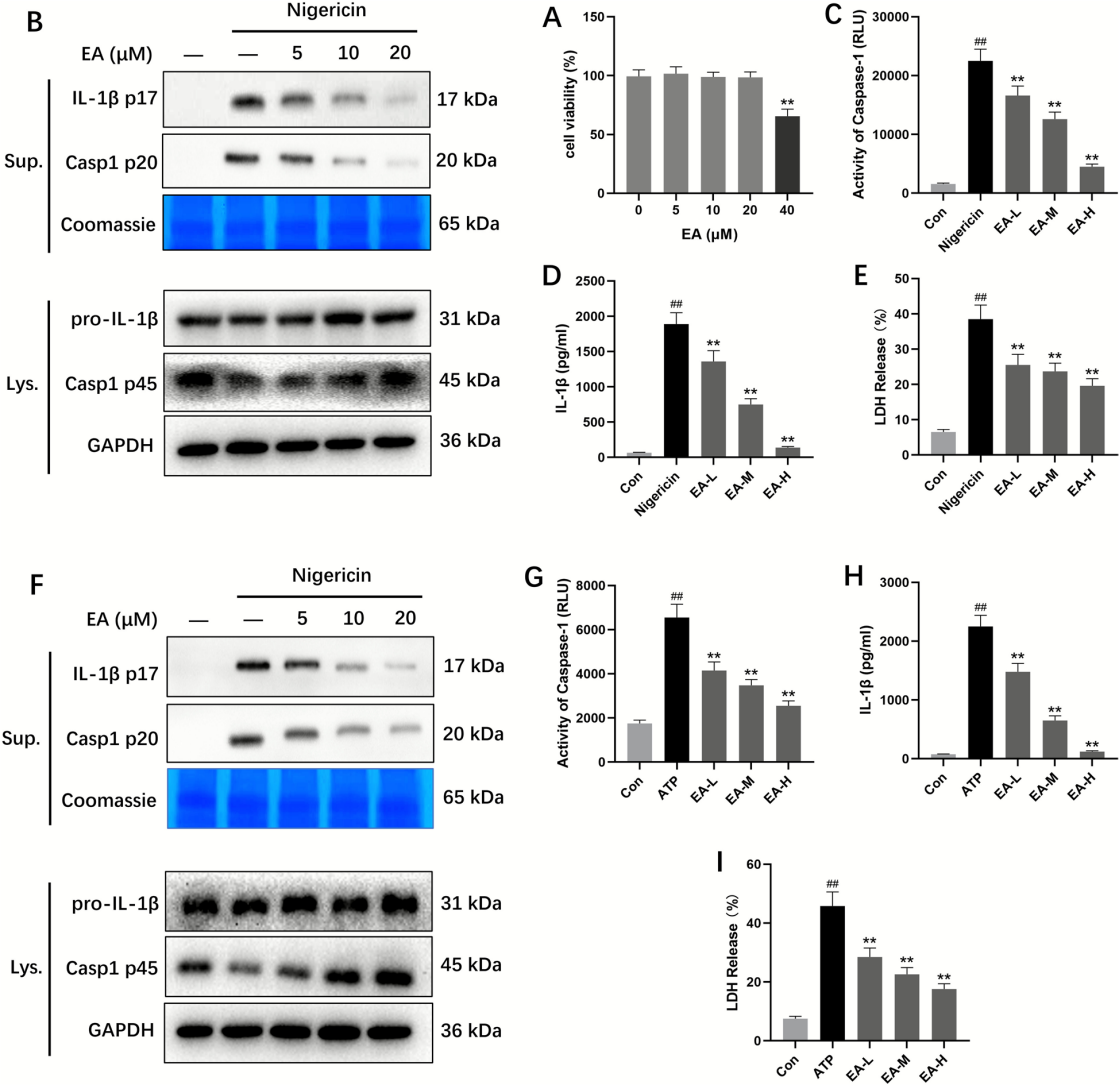
EA inhibited NLRP3 inflammasome activation in BMDMs and KCs. **A** Cell viability of BMDMs treated with EA. **B** LPS‑primed BMDMs were treated with various doses of EA for 1 h before stimulation with nigericin for 30 min. Western blotting was used to detect cleaved caspase‑1 and IL‑1β in the cell supernatants (Sup.) and the expression of caspase‑1 p45 and pro‑IL‑1β in the cell lysates (Lys.). **C** The activity of caspase‑1. **D** Secretion of IL‑1β. **E** Release of LDH. **F** LPS‑primed KCs were treated with various doses of EA for 1 h before stimulation with nigericin for 30 min. Western blotting was used to detect cleaved caspase‑1 and IL‑1β in the Sup and the expression of caspase‑1 p45 and pro‑IL‑1β in the Lys. **G** The activity of caspase‑1. **H** Secretion of IL‑1β. **I** Release of LDH. Data were presented as the mean ± SD (n = 3). ^##^*P* < 0.01 vs. control; ^**^P < 0.01 vs. nigericin

To further explore the impact of EA on NLRP3 inflammasome activation, we assessed its cytotoxic effects in BMDMs. Our analysis revealed that EA concentrations above 40 μM demonstrated significant cytotoxic properties (Fig. 4A). Previous research indicates that NLRP3 inflammasome activation triggers the processing and release of pro-caspase-1 and pro-IL-1β precursors. Experimental data demonstrated EA's potent suppression of caspase-1 activation pathways, IL-1β processing mechanisms, and LDH secretion patterns (Fig. 4B–E). Parallel experiments in KC cells showed comparable inhibition of caspase-1/IL-1β production and nigericin-induced LDH release (Fig. 4F–I). Collectively, these findings demonstrate EA's effective suppression of NLRP3 inflammasome activation in LPS-primed BMDMs and KCs models under controlled experimental conditions.

### EA inhibited canonical and noncanonical NLRP3 inflammasome activation but does not affect NLRC4 and AIM2 inflammasome activation

**Fig. 5:**
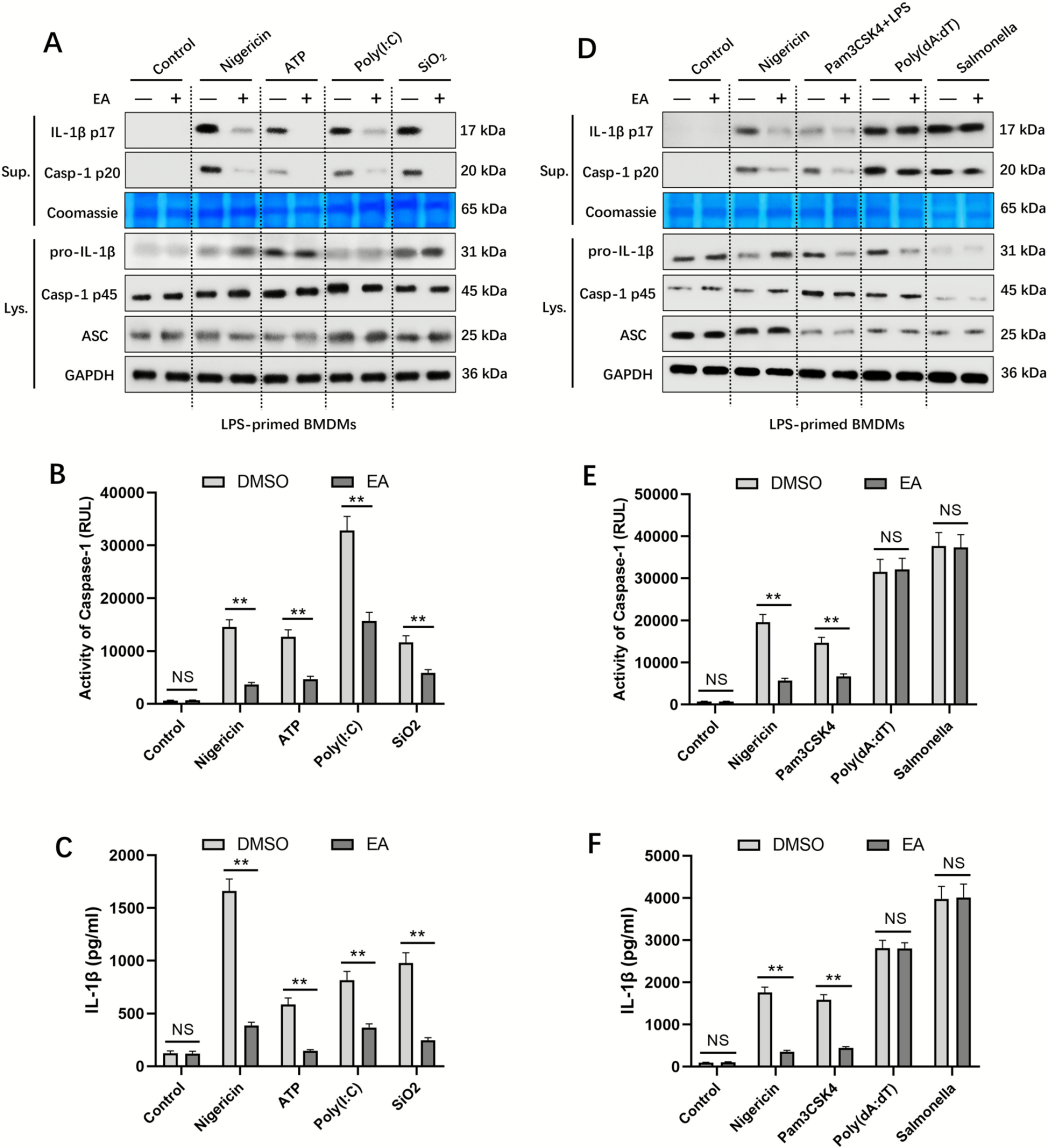
EA inhibited canonical and noncanonical NLRP3 inflammasome activation but has no effect on NLRC4 and AIM2 inflammasome activation. **A**–**C** LPS-primed BMDMs were incubated with or without EA (20 µM), followed by the indicated stimuli. Cleaved caspase-1(p20) and mature IL-1β (p17) in supernatant were assessed by western blotting, activity of caspase-1 and IL-1β secretion in the supernatants were analyzed. **D**–**F** LPS-primed BMDMs were incubated with or without EA (20 µM), followed by treatment with nigericin, poly (dA: dT) or Salmonella, or Pam3CSK4-primed BMDMs were treated with EA followed by transfection of LPS, cleaved caspase- 1(p20) and mature IL-1β (p17) were assessed by western blotting, activity of caspase-1 and IL-1β secretion in supernatants were evaluated. Data were presented as the mean ± SD (n = 3). ^**^P < 0.01; NS: not significant

The NLRP3 inflammasome can be triggered by both exogenous pathogen-derived molecules (PAMPs) and endogenous danger signals (DAMPs), including stimuli such as nigericin, SiO_2_, MSU, ATP, and poly (I: C) [13, 17, 20]. To explore this further, we examined EA's modulatory effects on NLRP3 activation pathways. Experimental data revealed that EA treatment significantly reduced caspase-1 levels and IL-1β release in BMDMs exposed to ATP, nigericin, SiO_2_, and poly (I: C) (Fig. 5A–C), demonstrating broad-spectrum inhibition across various NLRP3-activating stimuli. Furthermore, in Pam3CSK4-primed macrophages subjected to LPS transfection, EA effectively prevented caspase-1 proteolytic processing and maturation of IL-1β (Fig. 5D–F), indicating its capacity to suppress both canonical and alternative NLRP3 activation mechanisms. These collective findings establish EA as a broad NLRP3 inflammasome inhibitor.

The AIM2 and NLRC4 inflammasome complexes have been shown to facilitate caspase-1 activation pathways [33]. To evaluate EA's potential inhibitory effects, investigations were conducted on these alternative inflammasome mechanisms. Primed BMDMs were exposed to Salmonella typhimurium infection or poly (dA: dT) transfection to stimulate NLRC4 or AIM2 inflammasome activation respectively, following established methodologies [1, 15]. Notably, experimental data revealed that EA failed to impede either caspase-1 activation or IL-1β processing induced by these specific triggers (Fig. 5D–F). These findings demonstrate that EA selectively targets NLRP3 inflammasome activity without affecting AIM2 or NLRC4-mediated inflammatory pathways.

### EA suppressed the expression of NLRP3 protein and ASC oligomerization

**Fig. 6:**
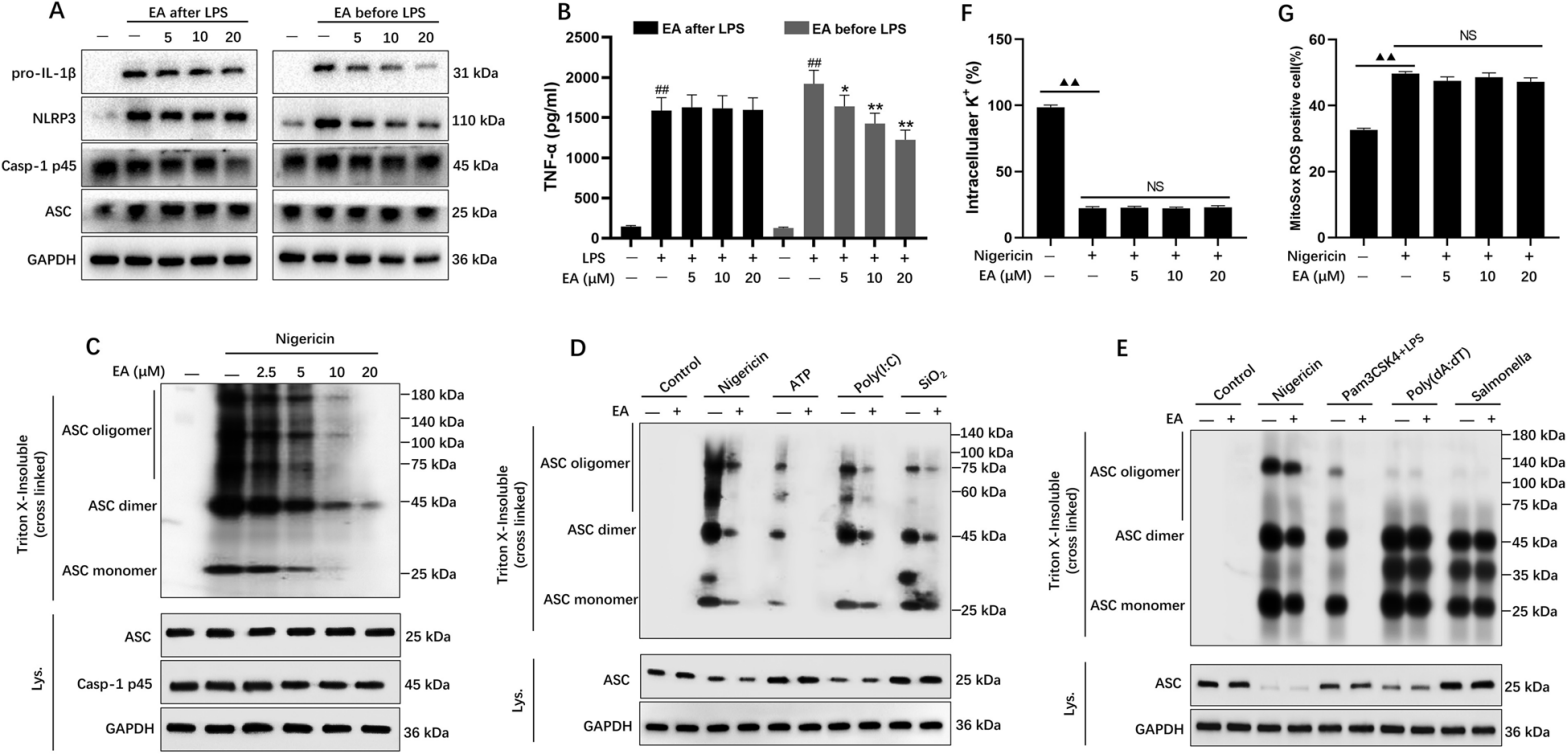
EA suppressed the expression of NLRP3 and blocked ASC oligomerization, but does not affect K^+^ or mitochondrial ROS production. **A** and **B** BMDMs were cultured with LPS for 4 h, then incubated with DMSO or EA for 1 h, or BMDMs were first incubated with EA for 1 h, followed by LPS treatment for 4 h, protein level of NLRP3 and pro-IL-1β was determined by western blotting, ELISA was used to detect TNF-α secretion. **C**–**E** Western blotting of cross-linked pellets from LPS-primed BMDMs treated with EA before being stimulated by the indicated agonists. **F** BMDMs were primed with LPS followed by EA incubation, and then treated with nigericin, the efflux of potassium was measured. **G** LPS-primed BMDMs were cultured in the presence or absence of EA and then stimulated with nigericin, then stained with MitoSox, mtROS was analyzed by flow cytometry. Data were presented as the mean ± SD (n = 3). ^##^*P* < 0.01 vs. control; ^**^P < 0.01 vs. LPS; ^▲▲^*P* < 0.01; NS: not significant

To examine EA's effects on LPS-induced NLRP3 and pro-IL-1β expression, we conducted sequential EA administration experiments using BMDMs. Cellular pretreatment with EA for 60 min prior to LPS exposure demonstrated significant suppression of both NLRP3 and pro-IL-1β, and the production of TNF-α (Fig. 6A and B). This temporal intervention pattern suggests EA exerts regulatory effects on NLRP3 inflammasome activation through protein-level inhibition, as evidenced by reduced NLRP3 activation markers in pretreatment conditions compared to post-stimulation administration.

ASC oligomerization plays a critical role in facilitating caspase-1 activation during inflammasome assembly, as documented in reference [32]. Experimental results demonstrated that EA dose-dependently suppressed nigericin-induced ASC oligomerization (Fig. 6C). Extended analysis revealed similar inhibitory effects on ASC aggregation when using alternative NLRP3 activators (Fig. 6D and E). Notably, EA exhibited specificity by showing no interference with ASC oligomerization processes in NLRC4 or AIM2 inflammasome pathways (Fig. 6E).

Subsequent analyses focused on elucidating the pathways through which EA modulates NLRP3 inflammasome stimulation, particularly examining potassium ion flux and reactive oxygen species formation [16, 26]. The initial phase of our investigation assessed EA's impact on cellular potassium dynamics. Nigericin stimulation was observed to markedly reduce intracellular potassium concentrations, though EA pretreatment showed no significant alteration in this process. This finding suggests EA's inhibitory effect targets NLRP3 activation mechanisms rather than influencing potassium efflux pathways (Fig. 6F). Parallel examination of oxidative stress markers revealed consistent ROS levels in EA-pretreated BMDMs across various stimulants including nigericin, poly (dA: dT), and Salmonella typhimurium challenges (Fig. 6G). These collective observations indicate EA likely exerts regulatory control over NLRP3 activation mechanisms occurring subsequent to potassium efflux and ROS generation events.

### EA repressed TGF‑β1 induced HSC activation via the YAP pathway

**Fig. 7:**
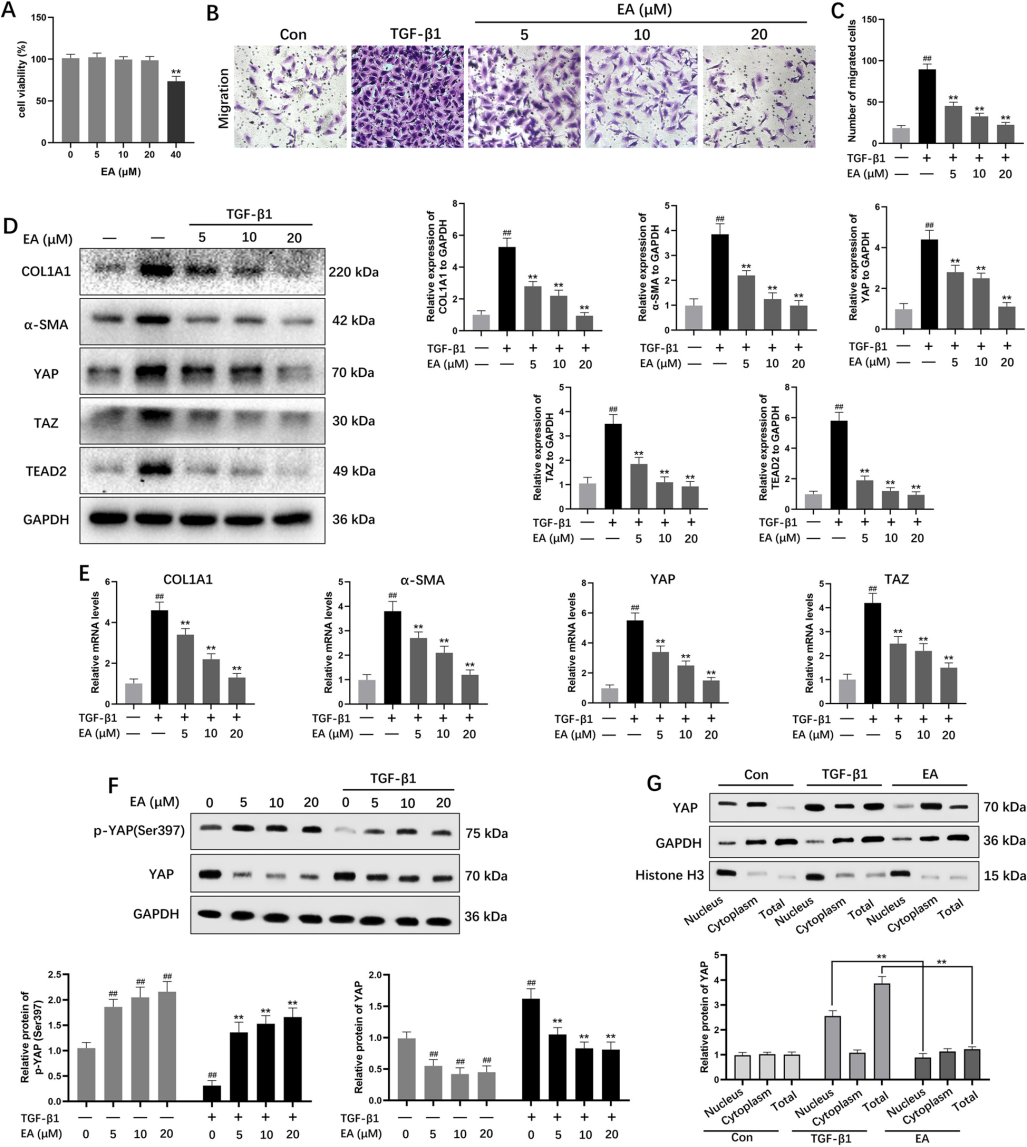
EA repressed TGF-β1 induced HSC activation through the YAP/TAZ pathway. **A** The cell viability of LX-2 treated by EA. **B** Cell migration was analyzed by transwell assay (scale bar: 50 μm). **C** The number of migrated cells. **D** The protein expression levels of YAP, TAZ, TEAD2, α-SMA and COL1A1 were markedly decreased by EA in LX-2 cells. **E** EA downregulated the mRNA expression of YAP, TAZ, α-SMA and COL1A1. **F** The protein expression level of p-YAP (Ser 397). **G** Protein expression of YAP in nucleus and cytoplasm. Data were presented as the mean ± SD (n = 3). ^##^*P* < 0.01 vs. control; ^**^P < 0.01 vs. TGF-β1

EA demonstrated a concentration-dependent suppression on the enhanced migratory activity of LX-2 HSCs induced by TGF-β (Fig. 7B and C). The YAP signaling cascade plays a critical regulatory role in HSC activation mechanisms. During early HSC activation, YAP undergoes nuclear translocation where it binds to multiple transcription factors, upregulating downstream target gene expression. Activation of the YAP/TAZ cascade promotes hepatic fibrogenesis through modulation of myofibroblast activity. To investigate EA's inhibitory effects on HSC activation through the YAP/TAZ axis, LX-2 cells were stimulated with TGF-β1 following administration of different EA concentrations. TGF-β1 exposure significantly increased YAP, TAZ, and TEAD2 expression in LX-2 cells. EA treatment substantially decreased both mRNA levels (COL1A1, α-SMA, YAP, TAZ) and protein expression (COL1A1, α-SMA, YAP, TAZ, TEAD2) as shown in Fig. 7D and E.

As illustrated in Fig. 7F, experimental data revealed that EA administration enhanced phosphorylation levels of YAP at the Ser397 site. This post-translational modification triggers proteasomal degradation of YAP, as confirmed through subsequent biochemical analyses. Current studies emphasize that subcellular localization of YAP, particularly its nuclear accumulation, serves as a key regulator of transcriptional activity. Subcellular fractionation analysis revealed predominant YAP localization in cytoplasmic fractions under baseline conditions. Notably, EA intervention demonstrated dual regulatory effects by suppressing total YAP protein levels and impairing its nuclear translocation capacity, as evidenced by comparative analysis of cytoplasmic and nuclear fractions (Fig. 7G).

**Fig. 8:**
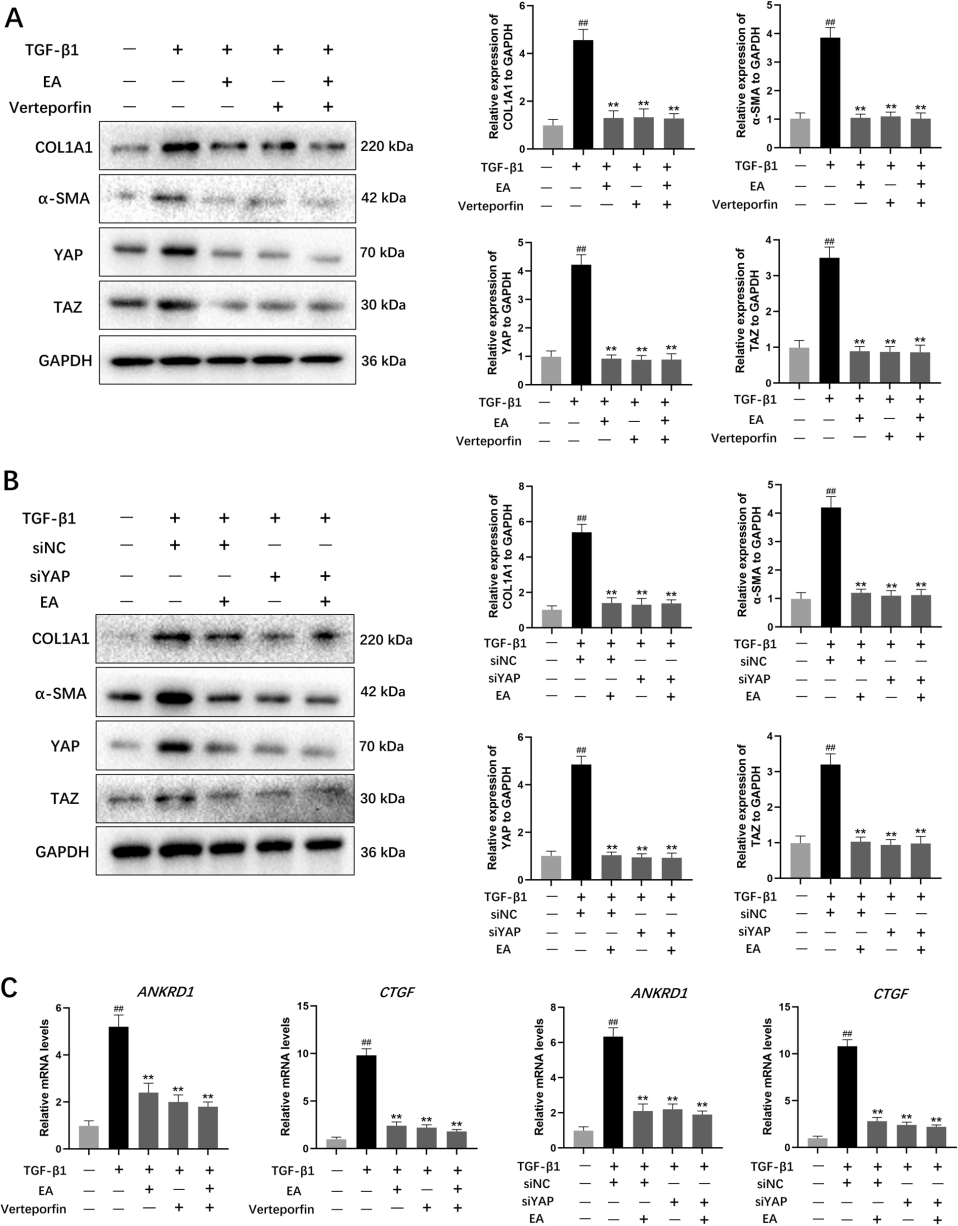
EA mediated inhibition of HSC activation depends on regulation of the YAP/TAZ pathway. **A** LX-2 cells were treated with EA (20 μM) and/or verteporfin, and COL1A1, α-SMA, YAP and TAZ protein expression was detected by western blot analysis. **B** YAP siRNA and EA (20 μM) were used to treat LX-2 cells, followed by WB analysis of the protein expression of COL1A1, α-SMA, YAP and TAZ. **C** LX-2 cells were treated with EA (20 μM), either alone or in combination with YAP siRNA and verteporfin. Then, the mRNA levels of CTGF and ANKRD1 were measured by qRT-PCR. Data were presented as the mean ± SD (n = 3). ^##^*P* < 0.01 vs. control; ^**^P < 0.01 vs. TGF-β1

To explore the potential involvement of YAP/TAZ signaling in EA-mediated attenuation of liver fibrosis, we administered VP alongside EA in LX-2 cell cultures. Figure 8A demonstrates that 1 μM VP notably suppressed TGF-β1-induced expression of YAP, TAZ, α-SMA, and COL1A1. However, combined administration of EA and VP did not effectively reduce the expression of these markers. Subsequent genetic silencing experiments revealed that YAP depletion substantially decreased YAP, TAZ, α-SMA, and COL1A1 levels in LX-2 cells, though EA treatment alone showed no significant effect on α-SMA and COL1A1 suppression (Fig. 8B). Notably, EA exposure caused marked downregulation of CTGF and ANKRD1—established YAP—responsive genes—in these cells. Neither YAP inhibition nor its genetic knockdown enhanced the EA-induced reduction of YAP or its transcriptional targets (Fig. 8C). Collectively, these experimental results indicate that EA exerts its anti-fibrotic effects on hepatic stellate cells primarily through regulation of YAP/TAZ-mediated signaling mechanisms.

### EA targeted the NLRP3 and YAP proteins

**Fig. 9:**
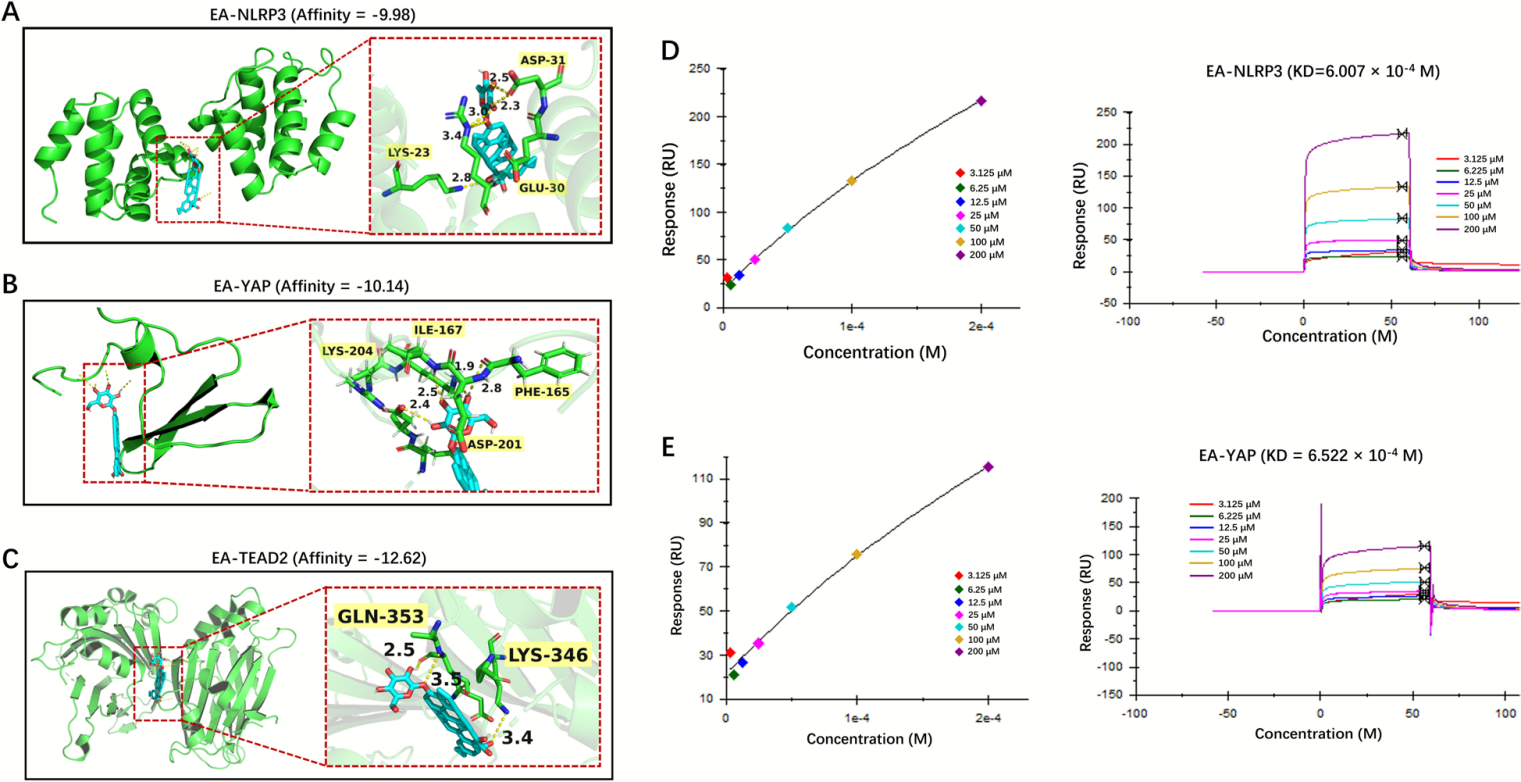
NLRP3 and YAP were the key targets of EA. **A** The schematic diagram of the molecular docking of EA with NLRP3, shown as the 3D diagram. The ribbon and stick structure displays the predicted bonds between EA and NLRP3. **B** The schematic diagram of the molecular docking of EA with YAP, shown as the 3D diagram. The ribbon and stick structure displays the predicted bonds between EA and YAP. **C** The schematic diagram of the molecular docking of EA with TEAD2, shown as the 3D diagram. The ribbon and stick structure displays the predicted bonds between EA and TEAD2. **D** Response value of interaction between EA and NLRP3 on CM5 chip. **E** Response value of interaction between EA and YAP on CM5 chip

To validate EA's protein interactions (NLRP3 and YAP), molecular docking and surface plasmon resonance (SPR) analyses were employed. Computational modeling identified optimal binding conformations for EA in NLRP3, achieving a docking score of −9.98 kcal/mol⁻^1^. EA's epoxide group formed a crucial hydrogen bond with LYS-23, ASP-31 and GLU-30 residues (Fig. 9A). EA was capable of forming hydrogen bonds with the residues LYS-204, ILE-167, PHE-165 and ASP-201 of YAP (−10.14 kcal/mol⁻^1^) (Fig. 9B). Parallel analysis demonstrated that EA could form hydrogen bonds with TEAD2 residues (GLN-353 and LYS-346), resulting in a binding energy of −12.62 kcal/mol. (Fig. 9C). SPR quantification using immobilized proteins showed NLRP3 bounded to EA with a dissociation constant of 6.007 × 10^−4^ M, and YAP bounded to EA with a dissociation constant of 6.552 × 10^−4^ M (Fig. 9D and E). The aforementioned studies had demonstrated that EA exhibited a significant binding affinity with proteins (NLRP3 and YAP).

## Discussion

Our research demonstrated that EA significantly improved critical pathological features including hepatic steatosis, inflammatory infiltration, and collagen deposition in MASH progression. Mechanistically, EA administration effectively blocked the advancement of MAFLD into MASH by suppressing inflammatory responses and fibrotic processes, potentially through dual modulation of both NLRP3 inflammasome activation and YAP signaling cascade. However, this study has certain limitations. This study did not validate the role of NLRP3 and YAP signaling pathways in mediating the therapeutic effects of EA on MASH using gene knockout mice or pharmacological inhibitors/activators. The other pathways enriched by liver RNA sequencing were not experimentally validated, limiting a comprehensive understanding of the overall effects of EA. Molecular docking and SPR experiments provided docking results but did not include in-depth validation of the binding sites. Meanwhile, this study lacks pharmacokinetic and safety data for EA; therefore, EA remains an experimental candidate compound, which currently limits its clinical applicability and translational potential.

Selecting an appropriate model is crucial for elucidating the pathogenesis of MASH and formulating preventive strategies. High-fat diet (HFD)-fed murine models are commonly employed to study MAFLD, typically resulting in obesity with fatty liver but lacking steatohepatitis and hepatic fibrosis [19]. The high-fat high-cholesterol (HFHC) diet is preferred for developing animal models of MASH due to its close resemblance to human MASH and its efficacy in facilitating the progression from nonalcoholic fatty liver to MASH, surpassing HFD alone [21]. Although the methionine-choline-deficient (MCD) diet-induced MASH model is widely utilized and reproducible, it does not fully replicate human MASH and fails to demonstrate elevated serum levels of triglycerides, cholesterol, insulin, glucose, and leptin [8, 41]. MCD models are typically employed for nonobese MASH studies [2]. Consequently, we utilized two murine models induced by MCD and HFHC diets to evaluate the therapeutic effects of EA. The findings revealed dose-dependent improvements in MASH pathology following EA treatment, evidenced by normalized liver enzyme profiles (AST/ALT), diminished lipid accumulation, reduced inflammatory cell infiltration, and attenuated fibrotic indicators. At molecular level, EA administration markedly suppressed both gene expression and protein abundance of NLRP3 inflammasome components (NLRP3, caspase 1, IL-1β) along with Hippo pathway effectors (YAP, TAZ, TEAD2). However, there are certain limitations regarding the selection of cell models in this study. Only the TGF-β1-induced LX-2 cell model was used for in vitro experiments. The absence of validation in primary hepatic stellate cells limits the generalizability of the findings.

Our investigation revealed that Ecliptasaponin A (EA) suppresses both NLRP3 and pro-IL-1β expression in bone marrow-derived macrophages (BMDMs) when administered prior to LPS priming. This evidence indicates EA's capacity to inhibit NLRP3 inflammasome formation by targeting the LPS priming phase (Fig. 4D). Experimental data demonstrated EA's disruptive effect on ASC oligomerization, effectively preventing NLRP3-ASC inflammasome complex assembly (Fig. 5A, B). Notably, EA maintained normal function in upstream NLRP3 activation pathways involving potassium ion efflux and reactive oxygen species generation. Specificity analysis showed EA's selective inhibition of NLRP3 without affecting AIM2 or NLRC4 inflammasomes (Fig. 4B). The molecular docking and SPR experiment further confirmed that EA exhibits a significant binding affinity for NLRP3. It was suggested that repressive activity of EA was dependent on NLRP3 protein.

Hepatic stellate cell (HSC) activation is critical for the progression of liver fibrosis [39]. YAP is activated early during HSC activation and serves as a downstream effector of the Hippo pathway [11]. Inhibition of YAP is crucial for maintaining HSC quiescence due to its significant role in HSC activation. Elevated YAP levels can sustain cellular activation states and stimulate extracellular matrix (ECM) production, worsening fibrotic progression. Following nuclear translocation, YAP initiates transcriptional regulation of Ctgf and Ankrd1, accelerating HSC activation dynamics. Increased Ctgf expression has been documented in fibrotic liver tissues and activated HSCs, promoting ECM protein biosynthesis and secretion [9]. Our investigation demonstrated that YAP silencing failed to enhance EA's inhibitory actions on HSC activation or fibrotic protein expression. VP treatment reduced YAP/TAZ levels post-TGF-β1 induction and diminished fibrosis indicators, indicating its therapeutic promise for hepatic fibrosis [35]. Notably, VP did not potentiate EA's suppressive effects on HSC activation or fibrotic protein expression patterns. Ctgf and Ankrd1 exhibited comparable expression profiles across experimental conditions. Through molecular docking and SPR analysis, we validated YAP as a direct molecular target of EA. These findings collectively indicate that EA's antifibrotic properties in MASH murine models likely operate through selective modulation of the YAP/TAZ signaling axis. This study demonstrates that EA exerts anti-inflammatory and anti-fibrotic effects in the MASH animal model through targeted modulation of NLRP3 and YAP signaling pathways.

## Conclusion

The experimental results demonstrated that EA effectively counteracted MASH development caused by MCD or HFHC dietary patterns through multi-target mechanisms. Specifically, EA administration significantly reduced pathological manifestations including steatosis, inflammatory infiltration, and collagen deposition in hepatic tissues. Mechanistic investigations revealed these therapeutic effects were mediated through dual-pathway regulation involving NLRP3 inflammasome deactivation and YAP signaling axis suppression. This pharmacological evidence supports EA's viability as a promising therapeutic agent for clinical management of metabolic dysfunction-associated steatohepatitis.

## Supplementary Information

Supplementary material 1.

## Data Availability

The data that support the ﬁndings of this study are available from the corresponding author upon reasonable request.

## Abbreviations

MASH: Metabolic dysfunction-associated steatohepatitis
MASLD: Metabolic dysfunction-associated steatotic liver disease
ALT: Alanine aminotransferase
AST: Aspartate aminotransferase
TC: Total cholesterol
TG: Triglycerides
EA: Ecliptasaponin A
NLRP3: NOD-like receptor protein 3
LPS: Lipopolysaccharide
BMDMs: Bone marrow-derived macrophages
KCs: Kupffer cells
HSCs: Hepatic stellate cells
TGF-β1: Transforming growth factor β1
YAP: Yes-associated protein
