# Supplementary material for: Ecliptasaponin A alleviates inflammation and fibrosis in experimental MASH mice via targeting the NLRP3 inflammasome and YAP signaling pathway
Source: Chin Med. 2026 Jan 13;21:32. doi: 10.1186/s13020-025-01321-9 (PMC12797505; doi:10.1186/s13020-025-01321-9)
Supplement: Supplementary file 1 — Supplementary material 1. [file 13020_2025_1321_MOESM1_ESM.docx]

EA not only directly binds to NLRP3 and YAP proteins but also down-regulates their gene and protein expression levels. A thorough discussion of the potential interplay between these two mechanisms is essential for fully understanding the pharmacological effects of this compound. Direct binding and the regulation of expression are not mutually exclusive; rather, they may be temporally coordinated and functionally interconnected, potentially amplifying each other in a synergistic manner to collectively contribute to the robust biological activity of EA.

The immediate effect of direct binding represents a rapid and targeted intervention. The interaction between EA and its target proteins can swiftly modulate their functional activities. Specifically, the direct binding of EA to NLRP3 inhibits the assembly and activation of the NLRP3 inflammasome, thereby promptly blocking Caspase-1 cleavage, the maturation of IL-1β, and GSDMD-mediated pyroptosis. Similarly, direct binding to YAP may disrupt its interaction with transcriptional co-activators such as TEAD or alter its conformation, leading to the immediate suppression of downstream gene transcription (e.g., CTGF, ANKRD1). This direct modulation serves as the "primary driving force" behind the compound's pharmacological efficacy and is characterized by high specificity. In contrast, the long-term and amplifying effect of expression regulation constitutes a sustained and reinforcing action, enabling prolonged modulation through the down-regulation of mRNA and protein levels. Following the initial inhibition of YAP transcriptional activity by EA, one potential downstream target could be YAP itself or a positive regulator within the pathway. This may initiate a negative feedback loop, resulting in reduced transcription and synthesis of YAP. Moreover, small-molecule binding can induce conformational changes in a protein, rendering it more susceptible to post-translational modifications such as ubiquitination, which promotes proteasomal degradation. Consequently, EA may not only inhibit the functions of YAP and NLRP3 but also accelerate their degradation, leading to a progressive decline in protein abundance.

In summary, the mechanism of action of EA involves a multi-step and mutually reinforcing process: initial direct binding and functional inhibition act as the triggering event, which subsequently induces cellular negative feedback regulation and alterations in protein stability, ultimately resulting in sustained down-regulation of target expression. The dual action of "functional inhibition" and "expression down-regulation" ensures a profound, durable, and synergistic suppression of the NLRP3 and YAP signaling pathways. Therefore, these two mechanisms should not be considered in isolation but instead reflect the sophisticated design of EA as a natural product in achieving highly efficient pharmacological activity. In future studies, we will further validate this integrated model experimentally, for example by measuring protein half-life to assess enhanced degradation or employing reporter gene assays to monitor feedback regulation of pathway activity. The above content will be included in the supplementary materials for presentation.
